# Risk prediction models for prolonged mechanical ventilation following coronary artery bypass grafting surgery: a systematic review and meta-analysis

**DOI:** 10.3389/fcvm.2025.1616003

**Published:** 2025-09-12

**Authors:** Yeru Jia, Zhiyi Pei, Xiaoxin Zhang, Chen Zhang, Xiaofeng Kang

**Affiliations:** ^1^ School of Nursing, Chinese Academy of Medical Sciences and Peking Union Medical College, Beijing, China; ^2^ Department of Nursing, Fuwai Hospital, National Center for Cardiovascular Diseases, Chinese Academy of Medical Sciences and Peking Union Medical College, Beijing, China

**Keywords:** coronary artery bypass grafting, prolonged mechanical ventilation, risk prediction model, meta-analysis, systematic review

## Abstract

**Objective:**

Prolonged mechanical ventilation (PMV) results in significant morbidity, mortality, and associated hospital costs. Models predicting PMV following Coronary artery bypass grafting (CABG) surgery were growing. However, the reliability, validity and clinical applicability of these models remain unclear. This systematic review and meta-analysis aim to provide a comprehensive quality assessment of PMV-risk prediction models for patients after CABG.

**Methods:**

Nine relevant domestic and international databases were systematically searched from inception until November 4, 2024 using PICOTS format. The Prediction Model Risk of Bias Assessment Tool (PROBAST) checklist was employed to evaluate the risk of bias and applicability of each study. A meta-analysis of the area under the curve (AUC) values from model external validations was conducted using R software.

**Results:**

Fifteen studies detailing 12 PMV-risk prediction models were included, with AUC values ranging from 0.561 to 0. 875. In the meta-analysis, the pooled AUC was 0.696 (95% CI: 0.553, 0.839, I-squared = 90.4%) for externally validated studies of three Society of Thoracic Surgeons (STS) models. The most frequently used predictors in the models were grouped into demographics, medical history, examination, and supportive therapy.

**Conclusions:**

Although studies were judged as high overall risk of bias according to PROBAST guidelines evidence from our review indicates that risk factors of PMV in Post CABG Patients include age, BMI, history of cardiac surgery, history of cardiovascular disease, COPD, EF/LVEF, IABP, and cardiopulmonary bypass.

**Systematic Review Registration:**

PROSPERO CRD42024608639.

## 1 Introduction

Coronary artery disease (CAD) remains a critical global health issue, with epidemiological studies up to 2022 revealing a staggering 315 million cases worldwide (1). Significant regional disparities exist in age-standardized CVD mortality rates, ranging from 73.6 per 100,000 population in high-income Asia-Pacific regions to 432.3 per 100,000 population in Eastern Europe (2). Coronary artery bypass grafting (CABG) stands as the most frequently performed cardiac surgery. Epidemiological data show an annual CABG rate of 36.7 procedures per 100,000 population (3). In the perioperative period, mechanical ventilation plays an indispensable role in stabilizing patients and aiding their recovery (4). However, prolonged mechanical ventilation (PMV) following CABG is associated with severe complications such as pulmonary injury, pneumonia, muscle atrophy, functional impairment, and diaphragmatic dysfunction, which significantly compromise patient safety and escalate healthcare costs. The prevalence of PMV has been reported to reach up as high as 48.11% (5, 6). Therefore, multiple studies focus on the accurate prediction and intervention for PMV risk to improve clinical outcomes and reduce complications (7, 8).

The definition of PMV typically refers to the need for continued mechanical ventilation beyond a certain duration following CABG (9), while the PMV time varied more than 12, 24, 48, or 72 h. This heterogeneity complicates cross-study comparison and underscores the challenge of reliable prediction. Existing research has identified several patient- and procedure-related risk factors, including advanced age, reduced left ventricular ejection fraction (LVEF), higher New York Heart Association (NYHA) class (10), chronic obstructive pulmonary disease (COPD), prolonged cardiopulmonary bypass time (11), and elevated inflammatory markers (12). In parallel, prediction models based on statistical or machine-learning methods have been developed to integrate these variables and provide individualized risk estimates. For instance, risk-scoring models developed by Dallazen-Sartori et al. (13) and Fan et al. (14) demonstrated good predictive performance. Widely used clinical tools, such as the Society of Thoracic Surgeons (STS) risk model and EuroSCORE, offer perioperative risk references but are not tailored to PMV after CABG, which may limit precision (9). Although CABG-specific scoring systems have been proposed, most are single-center, adopt heterogeneous PMV definitions, report variable discrimination and inconsistent calibration, and lack robust external validation (15). Collectively, these limitations mean that the current evidence remains conflicting, which justifies a rigorous synthesis and appraisal of available models.

A recently published review examined existing prediction models for PMV in CABG patients and identified a critical limitation: the absence of quantitative summary assessments of model performance (16). This shortcoming impedes a comprehensive and intuitive understanding of the overall predictive efficacy of these models. To address this gap, the present study systematically reviews and evaluates current risk prediction models for PMV in CABG patients, with a focus on methodological quality assessed by the PROBAST tool and pooled performance through meta-analysis. The findings are expected to offer valuable guidance for clinical practice by enabling early identification of high-risk individuals and supporting timely, evidence-based preventive interventions.

## 2 Materials and methods

The study protocol has been registered with PROSPERO (registration number: CRD42024608639). Compliance with the TRIPOD-SRMA guidelines ensures methodological transparency and rigor in the execution of systematic reviews and meta-analyses of prediction model studies.

### 2.1 Literature search

Nine databases (China National Knowledge Infrastructure (CNKI), Wanfang Database, China Science and Technology Journal Database (VIP), SinoMed, PubMed, Web of Science, The Cochrane Library, Cumulative Index to Nursing and Allied Health Literature (CINAHL), and Embase) were searched from their inception to November 4, 2024. The detailed search strategy was presented in Supplementary Material 1.

This systematic review was guided by the PICOTS framework, in alignment with the recommendations of the CHARMS checklist for the appraisal and data extraction of prediction modeling studies (17). The PICOTS framework was used to define the review's objectives, formulate the search strategy, and establish the inclusion and exclusion criteria for the studies (9, 18). The detailed PICOTS specification is provided in Supplementary Material 2.

### 2.2 Selection of studies

The inclusion criteria for studies were as follows: (1) studies involving patients who have undergone CABG; (2) study participants aged 18 years or older; (3) observational study design; (4) studies focused on the development and validation of risk prediction models for PMV following CABG.

The exclusion criteria were: (1) studies that analyze risk factors without developing a risk prediction model; (2) studies with incomplete data or inaccessible original texts; (3) conference abstracts, study protocols, duplicate publications, and studies that do not report the specified outcomes.

### 2.3 Study selection and screening

Titles, abstracts, and full texts were independently assessed for eligibility by two reviewers (JYR and PZY), and reference lists of selected studies were screened to identify further relevant literature. Any discrepancies were resolved through consensus in consultation with a third reviewer (ZXX). All researchers are familiar with the predictive modeling methodology and have received training in evidence-based nursing.

### 2.4 Data extraction

Two reviewers (JYR and PZY) independently assessed the retrieved records and determined full-text eligibility. Conflicts were resolved through consensus or by arbitration from a third reviewer (ZXX). The researchers employed the CHARMS checklist to create a data extraction form for recording the literature's characteristics. The extracted data from the included studies were organized into two categories: (1) Basic study characteristics, including author, publication year, study design, data source, and sample size; (2) Prediction model details, such as variable selection approach, model development technique, validation type, performance metrics, handling of missing data, treatment of continuous variables, final predictors, and model presentation format.

### 2.5 Quality assessment

Three reviewers (JYR, PZY, and ZXX) independently assessed the quality and risk of bias of the included studies using the PROBAST tool, which evaluates the risk of bias and applicability of multivariable prediction models for diagnosis or prognosis. This review specifically targeted prognostic models predicting PMV in CABG patients. The PROBAST tool consists of 20 items across four domains: participants, predictors, outcomes, and analysis. Each domain contains signaling questions rated as “Yes,” “Probably yes,” “Probably no,” “No,” or “No information”, with a “Yes” indicating no bias. Applicability concerns are assessed in the first three domains, and the overall risk of bias is classified as low, high, or unclear. Two reviewers (JYR and PZY) independently evaluated all articles and Supplementary Materials based on established criteria, resolving discrepancies through discussion or consultation with a third reviewer (ZXX).

### 2.6 Data synthesis and statistical analysis

R software (version 4.4.1) was used to conduct a meta-analysis of AUC values derived from validated models, with heterogeneity assessed through the *I*^2^ index. The *I*^2^ values of 25%, 50%, and 75% corresponded to low, moderate, and high heterogeneity, respectively. Fixed or random effects models were selected based on the heterogeneity of the analysis results. Publication bias was evaluated by visual inspection of funnel plots. As fewer than 10 studies were included, Egger's regression was not conducted (19).

## 3 Results

### 3.1 Study selection

**Figure 1:**
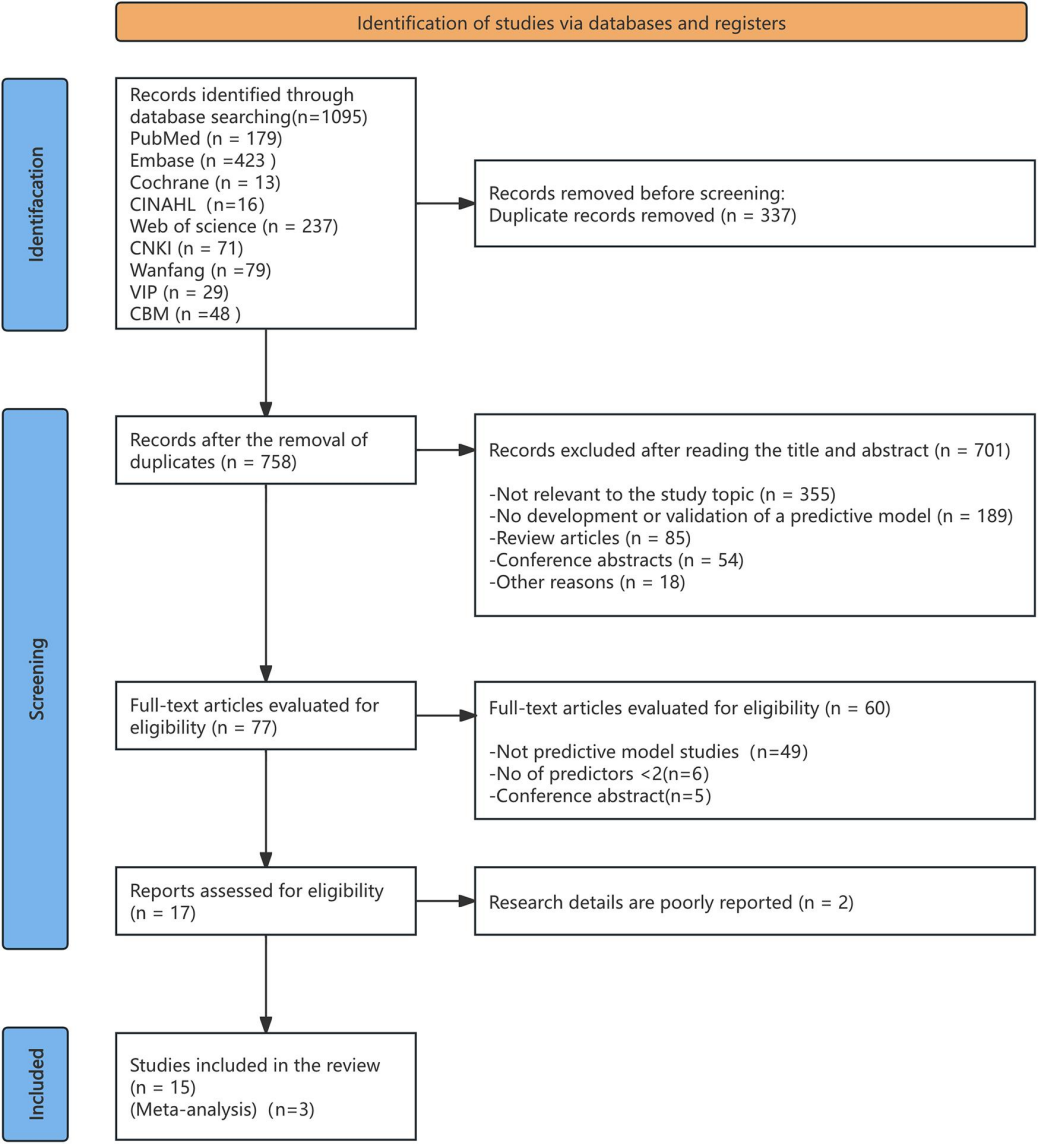
PRISMA flow diagram outlining the process of literature identification and selection for the systematic review.

Figure 1 displays the PRISMA 2020 flowchart, illustrating the search process and its outcomes. The initial search yielded 1,095 indexed records. After eliminating 337 duplicates, 758 titles and abstracts were reviewed for eligibility, resulting in the selection of 77 articles for further evaluation. Following evaluation, 701 studies were excluded due to inconsistent outcome indicators or research content, 49 studies were not related to model construction or validation, 6 studies had fewer than two predictors, and 5 were conference abstracts. Ultimately, 15 studies were included for detailed analysis.

### 3.2 Study characteristics

A total of 15 articles were included in this study, published between 1996 and 2024, with five studies published in the past five years. Geographically, studies were conducted in China (*n* = 5, including four in Chinese), the United States (*n* = 3), Brazil (*n* = 3), and one each from Canada, Italy, New Zealand, and Spain. Of the included studies, three were prospective and conducted at single centers, while the remaining twelve were retrospective, including one multicenter study. Most targeted the general CABG population; however, three focused on off-pump coronary artery bypass grafting (OPCABG) patients, and one excluded them. The sample sizes varied widely, from 110 to 439,092 participants, with a total of 454,703 patients across all studies. Definitions of PMV varied across studies. Most adopted a threshold of >24 h of ventilation, while others applied shorter (e.g., >12 h) or longer (e.g., >48 h or more) cutoffs and a few studies reported multiple thresholds (24 h, 48 h, 72 h). Reported incidence ranged from 3.0% to 43.4% (Table 1).

**Table 1:** Summary of the fundamental characteristics of the included studies.

Author (year)	Country	Study	Model type	Date source	Events(n)/Total participants (*N*)	Definition
Habib et al. (20）	United States	Retrospective	Development	From St. Vincent Medical Center, Toledo, Ohio	15/507	>24 h
Légaré et al. (21)	Canada	Retrospective	Internal validation	From a single university affiliated institution	157/1,829	>24 h
Serrano et al. (22)	Spain​	Prospective	Internal validation	From an ICU in a tertiary referral hospital	24 h:59/569 48 h:43/569 72 h:36/569	>24, 48, 72 h
Cislaghi et al. (33)	Italy	Prospective	Development	From an ICU in Luigi Sacco Hospital	1,420/3,629	>12 h
Mu et al. (23)	China	Prospective	Internal validation	From Peking University First Hospital	18/243	>24 h
Ikeoka et al. (24)	Brazil	Retrospective	External validation	From the TotalCor Hospital, located in São Paulo	NR/659	>24 h
Wang et al. (25)	New Zealand	Retrospective	External validation	From Auckland City Hospital	108/818	>24 h
Mendes et al. (32)	Brazil	Retrospective	Internal validation	From the Federal University of São Carlos and the Hospital de Base of São José do Rio Preto	90/1,315	>24 h
Wise et al. (26)	United States	Retrospective	Internal validation	From Vanderbilt University Adult Cardiac Database	104/738	>24 h
O'Brien et al. (27)	United States​	Retrospective	Development; Internal validation	From the Society of Thoracic Surgeons Adult Cardiac Surgery Database (STS ACSD)	Derivation cohort: 40,974/4, 39,092	>24 h
Wang et al. (28)	China	Retrospective	Development; Internal validation; External validation	From a single center in Tianjin, Fourth Central Hospital	28/110	>24 h, （cumulative time)
Dallazen-Sartori et al. (13)	Brazil	Retrospective	Internal validation	From the Cardiac Surgery Postoperative Unit of Hospital São Lucas of PUCRS.	783/4,165	>12 h
Zhou et al. (29)	China	Retrospective	Internal validation	From the Department of Cardiovascular Surgery, General Hospital of Northern Theater Command	107/683	>24 h
Qi et al. (30)	China	Retrospective	Internal validation	From Ningbo First Hospital	26/96	>24 h
Liu et al. (31)	China	Retrospective	Internal validation	From Wuhan Asian Heart Hospital affiliated with Wuhan University of Science and Technology	52/250	>48 h

Across the 15 included studies, twelve prediction models were identified. One study reported three models (22), and another two studies reported two models respectively (26, 32). Most were developed using logistic regression, while a few also explored machine-learning approaches such as artificial neural networks (ANNs) (26, 32). Three studies focused on model development, whereas the majority reported internal validation, and only a small number underwent external validation. Discrimination was commonly assessed using the area under the curve (AUC), which showed moderate to good performance across studies. Calibration was less consistently reported, typically using the Hosmer–Lemeshow test, with only one study presenting calibration curves (27). Models were most often presented as simple scoring systems (13, 21–26, 30), although a few were provided as nomograms or equations (27, 29). Details of model development, validation, and performance are summarized in Table 2.

**Table 2:** Provides details of the prediction models reported in the included studies.

Author (year)	Missing data handling	Continuous variable processing method	Variable selection	Modeling method	Calibration method	Validation method	Model performance	Model presentation format
Habib et al. (20）	NA	Continuous variable	Backward elimination	Multivariate logistic regression;Multivariate linear regression	NA	NR	NA	NR
Légaré et al. (21)	Excluded	Categorical variable	Forward Stepwise Regression Analysis	Logistic regression	NA	Internal, Bootstrap	AUC = 0.81, 95%CI (0.7916–0.8284)	Risk scoring system
Serrano et al. (22)	NA	Categorical variable	NA	NA	Hosmer-Lemeshow	Internal	24h: AUC = 0.661, 95%CI (0.577–0.744) 48 h: AUC = 0.676, 95%CI (0.580–0.772) 72 h: AUC = 0.651, 95%Cl (0.551–0.752)	Risk scoring system
Cislaghi et al. (33)	Excluded	Categorical variables	Univariate analysis	Multivariate logistic regression	Hosmer-Lemeshow	NR	NA	NR
Mu et al. (23)	Excluded	Continuous variable	Univariate analysis	Multivariate logistic regression	NA	Internal	AUC = 0.693, 95% CI (0.582–0.803)	Risk scoring system
Ikeoka et al. (24)	NA	NR	NA	NA;	Hosmer-Lemeshow	External validation of STS model	AUC = 0.80, 95% CI (0.71–0.88）	Risk scoring system (Website calculator)
Wang et al. (25)	NA	NR	NA	Logistic regression	Hosmer-Lemeshow	External validation of STS model	AUC = 0.561, 95% CI (0.501–0.622)	Risk scoring system
Mendes et al. (32)	NA	Continuous variable	Univariate analysis	Logistic regression; artificial neural networks (ANNs)	Hosmer-Lemeshow	Internal, Cross-validation	logistic regression: AUC = 0.67,95%CI(0.57–0.78); artificial neural networks: AUC = 0.72,95% CI (0.64–0.81）	NR
Wise et al. (26)	Multiple imputation	Categorical variable	Forward stepwise selection	Logistic regression; Artificial neural networks (ANN)	NA	Internal, K-fold validation	logistic regression: AUC = 0.698,95%CI(0.648–0.748）; artificial neural networks: AUC = 0.732,95%CI (0.682–0.782）	Risk scoring system
O'Brien et al. (27)	Multiple imputation	Categorical variable	Backward stepward selection	Logistic regression	Calibration curves. Hosmer-lemeshow	Internal, split sample 6:4	AUC = 0.772, 95%CI (0.7708–0.7732)	Nomogram
Mendes et al. (32)	NA	Continuous variable	Univariate analysis	Logistic regression; artificial neural networks (ANNs)	Hosmer-Lemeshow	Internal, Cross-validation	logistic regression: AUC = 0.67,95%CI(0.57–0.78); artificial neural networks: AUC = 0.72,95% CI (0.64–0.81）	NR
Wise et al. (26)	Multiple imputation	Categorical variable	Forward stepwise selection	Logistic regression; Artificial neural networks (ANN)	NA	Internal, K-fold validation	logistic regression: AUC = 0.698,95%CI(0.648–0.748); artificial neural networks: AUC = 0.732,95%CI (0.682–0.782）	Risk scoring system
O'Brien et al. (27)	Multiple imputation	Categorical variable	Backward stepward selection	Logistic regression	Calibration curves. Hosmer-lemeshow	Internal, split sample 6:4	AUC = 0.772, 95%CI (0.7708–0.7,732)	Nomogram
Wang et al. (28)	NA	Categorical variable	Backward stepward selection	Logistic regression	Hosmer-Lemeshow	Internal; External validation of STS model	Internal cohort: AUC = 0.770, 95%CI (0.680–0.845) Validation group: AUC = 0.736, 95%CI (0.644–0.816)	Risk scoring system
Dallazen-Sartori et al. (13)	NA	Categorical variable	Backward stepward selection (logistic regression model)	Logistic regression	Hosmer-Lemeshow	Internal, Cross-validation	AUC = 0.66, 95%CI (0.64–0.68)	Risk scoring system
Zhou et al. (29)	NA	Categorical variables	Forward stepwise selection	Multivariate logistic regression	NA	Internal	AUC = 0.727, 95%CI (0.674–0.781)	Equation
Qi et al. (30)	NA	partially categorized	Univariate analysis	Multivariate logistic regression	NA	Internal	AUC = 0.875, 95%CI (0.792–0.934)	Risk scoring system
Liu et al. (31)	NA	Categorical variable	Forward stepwise multivariate logistic regression	Multivariate logistic regression	NA	Internal, Cross-validation	AUC = 0.805, 95%CI (0.718–0.892)	NR

### 3.3 Predictors included in the models

**Figure 2:**
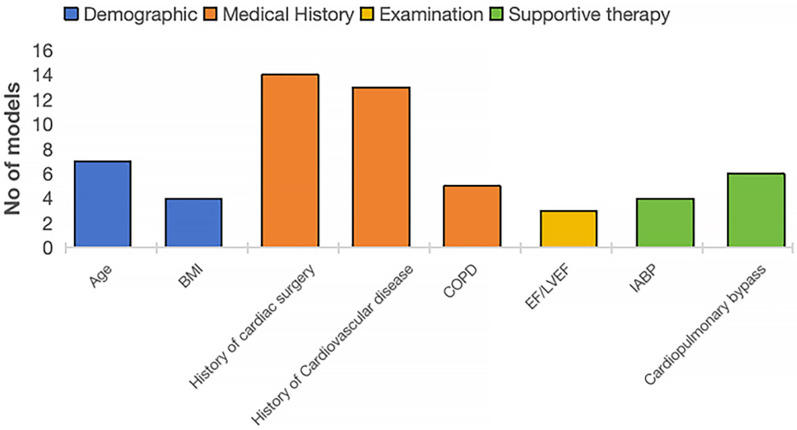
Predictors with higher frequency.

Predictors for PMV were diverse and encompassed demographic characteristics, medical history, examination results, and treatment approaches. The number of predictors retained in the final models varied considerably. When grouped into four domains (Figure 2), a few predictors consistently emerged as important, particularly history of cardiac surgery, cardiovascular disease, older age, chronic obstructive pulmonary disease, and use of cardiopulmonary bypass (Supplementary Material 3).

### 3.4 Results of quality assessment

**Figure 3:**
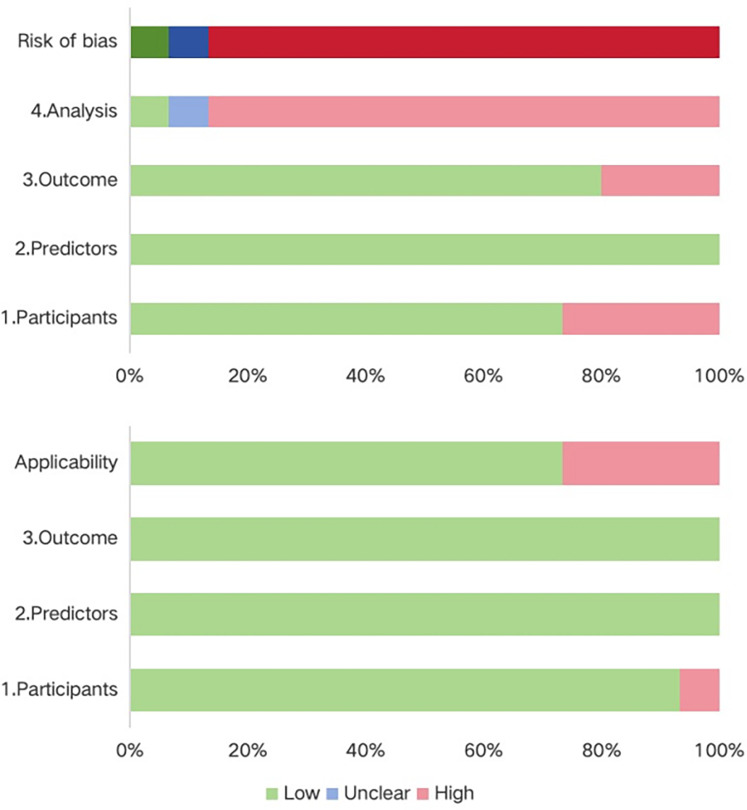
Summary of risk of bias and applicability evaluation (PROBAST).

Most studies were assessed as having a high risk of bias, underscoring methodological shortcomings in their development or validation processes. Within the participant domain, four studies were classified as high risk, primarily due to the use of inappropriate data sources (21, 28, 29, 31). Conversely, all studies demonstrated a low risk of bias in the predictor domain. In the outcome domain, bias mainly stemmed from inadequate or inconsistent definitions and assessment methods, with some studies lacking standardized criteria and relying on subjective measures (22, 29, 30). The analysis domain was the most problematic. Many studies suffered from insufficient sample sizes that fell below the recommended threshold of >20 events per variable (EPV) (22–26, 31, 33). In addition, continuous variables were frequently transformed into categories, often leading to information loss (9, 14, 15, 17, 18, 20, 21, 32). A number of models selected predictors solely through univariable analysis (23, 30, 32, 33), and several failed to apply strategies to mitigate overfitting (20, 26, 31). None reported on potential underfitting, optimism in performance estimates, or the complexity of the data structure. With respect to applicability, most models were judged as low risk, though a few were rated high risk in the participant domain because of inappropriate exclusions (21, 28, 29, 31). Predictors and outcomes were generally applicable, resulting in overall good clinical applicability. Details on the risk of bias and applicability assessments are shown in Figure 3; Table 3.

**Table 3:** Quality assessment (risk of bias and applicability) of the included studies based on the PROBAST tool.

Author (year)	ROB				Applicability			Overall	
	Participants	Predictors	Outcome	Analysis	Participants	Predictors	Outcome	ROB	Applicability
Dallazen-Sartori et al. (13)	+	+	+	?	+	+	+	?	+
Légaré et al. (21)	−	+	+	−	−	+	+	-	-
Ikeoka et al. (24)	+	+	+	−	+	+	+	-	+
Wang et al. (25)	+	+	+	−	+	+	+	-	+
Liu et al. (31)	−	+	+	−	+	+	+	-	-
Mendes et al. (32)	+	+	+	−	+	+	+	-	+
O'Brien et al. (27)	+	+	+	+	+	+	+	+	+
Serrano et al. (22)	+	+	−	−	+	+	+	-	+
Wang Ziyu et al. (28)	−	+	+	−	+	+	+	-	-
Wise et al. (26)	+	+	+	−	+	+	+	-	+
Mu Dongliang et al. (23)	+	+	+	−	+	+	+	-	+
Zhou Shicheng et al. (29)	−	+	−	−	+	+	+	-	-
Qi Yanqing et al. (30)	+	+	−	−	+	+	+	-	+
Cislaghi et al. (33)	+	+	+	−	+	+	+	-	+
Habib et al. (20)	+	+	+	−	+	+	+	-	+

ROB, risk of bias; +, indicates low ROB/low concern regarding applicability; −, indicates high ROB/high concern regarding application; ?, Indicates unclear ROB/unclear concern regarding applicability.

### 3.5 Meta-analysis of validation models included in the review

**Figure 4:**
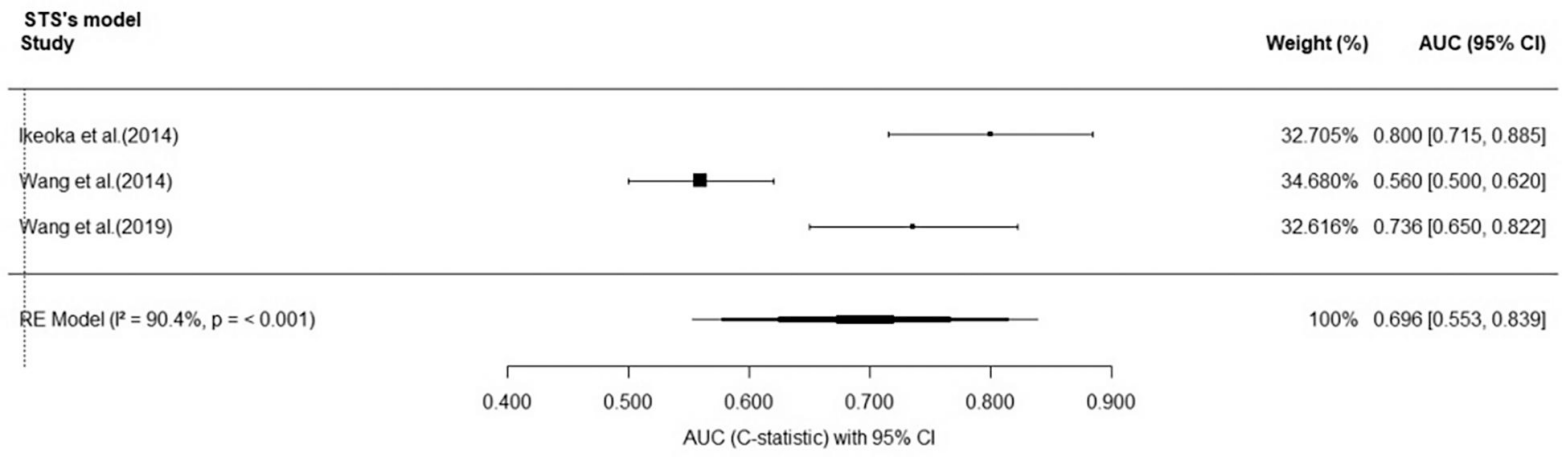
Forest plot showing the area under the receiver operating characteristic curve for the risk prediction model.

After screening, it was found that three articles were externally validated based on the STS scoring system. A total of 1,587 patients contributed to the meta-analysis. The pooled area under the receiver operating characteristic curve (AUC), representing the overall discriminative performance of the included prediction models, was 0.696 (95% CI: 0.553–0.839) based on a random-effects model (Figure 4). The *I*^2^ value was 90.4% (*p* < 0.001), indicating a high degree of heterogeneity among the studies. From a study design perspective, the high heterogeneity observed in the meta-analysis is likely attributable to differences in sample sizes and the handling of predictors among the STS studies. However, due to the limited number of available studies, detailed subgroup analyses could not be performed. Visual inspection of the funnel plot suggested a symmetrical distribution of studies, indicating potential significant publication bias (Supplementary Material 4). Additionally, sensitivity analysis confirmed the robustness of the results, as no single study significantly impacted the pooled effect size (Supplementary Material 5).

## 4 Discussion

This study evaluated the predictive models from 15 studies and found that these models demonstrated moderate to good predictive performance in both internal research and external validation. Notably, the PROBAST tool assessment indicated that the vast majority of the studies had a high risk of bias, suggesting that the current predictive models still need improvement in terms of design and reporting quality. Factors influencing the risk of bias may include heterogeneity in the modeled population, inconsistent selection of predictive factors, and differences in statistical methods. To the best of our knowledge, this study is the first systematic performance evaluation of predictive models for PMV risk in CABG patients. Our findings provide important references for clinicians and nurses, helping them to scientifically select and interpret risk assessment tools when identifying high-risk patients before surgery. Additionally, this study also offers directions and methodological inspirations for the development of high-quality predictive models in the future.

The inconsistent measurement of PMV has a significant impact on the interpretive power of risk models. Most models in this study used the STS definition, classifying PMV as postoperative ventilation of more than 24 h, while others used thresholds of over 12 or 48 h. A stricter definition, such as 48 h, may improve model discrimination, while a shorter threshold, like 12 h, increases sensitivity but may introduce heterogeneity affecting model accuracy. These definitional discrepancies hinder data integration across studies. Although the models in this study show robustness, different PMV definitions can limit their generalizability and application in various clinical settings. Future research may explore dynamic prediction models that combine real-time data during and after surgery to enhance predictive ability.

In this study, the number of predictive variables across models ranged from 3 to 51. However, excessive variable inclusion may hinder a model's practicality in clinical application. It is therefore preferable to adopt a streamlined set of clinically relevant and readily accessible predictors. Future research should focus on automating risk assessment through variable simplification or integration with electronic health record systems, thereby reducing operational complexity and improving clinical efficiency. Commonly reported predictors in existing studies can be categorized into four domains: demographic, medical history, examination, and supportive therapy. Among these, age and body mass index (BMI) are the most frequently cited demographic factors. Advanced age indicates reduced physiological reserve and multiple comorbidities, and has been almost consistently demonstrated to be a predictor of PMV (34, 35). Low BMI may correlate with weaker lung function and immunity, while high BMI with increased respiratory pressure and anesthetic challenges (35). Only one model uses high BMI (≥30 kg/m^2^) as a PMV predictor (13). Incorporating BMI stratification may improve predictive accuracy. Medical history factors, such as history of cardiac surgery, cardiovascular disease, and COPD, are frequent non-modifiable predictors impairing cardiac and pulmonary function. The ejection fraction (EF) is easily obtained and can facilitate preoperative risk assessment and aid in postoperative management decisions, however, there is no clear consensus on the threshold for EF (e.g., 30% or 50%) in predicting PMV risk, as data on an optimal cutoff are currently lacking. Supportive therapy is also a significant consideration. The use of an intra-aortic balloon pump (IABP) has been shown to strongly predict PMV risk and is often used both intraoperatively and preventively in CABG or OPCABG patients (36). Additionally, it can serve as a protective factor. The use of cardiopulmonary bypass increases hemodynamic and respiratory strain, triggering a systemic inflammatory response syndrome, which can lead to PMV postoperatively. Opting for OPCABG may reduce the incidence of PMV in certain patients, although this benefit depends on individual patient characteristics and the surgeon's proficiency with the technique. These predictors are widely recognized as PMV risk factors in literature and practice. Combining predictive model results with preoperative and postoperative interventions may improve high-risk patient prognosis. For example, for patients with high BMI identified preoperatively, the incidence of postoperative pulmonary complications can be reduced through preoperative respiratory function training and nutritional management. Future research should focus on evaluating the effectiveness of these interventions to achieve more precise risk management. At the same time, with the continuous advancement of technology (such as minimally invasive coronary artery bypass grafting), it is expected to reduce surgical trauma, shorten mechanical ventilation time, and accelerate recovery. In this study, almost half of the models included were developed or validated using data from the Chinese population, followed by the United States. This trend suggests that PMV risk prediction in CABG patients is gaining increasing attention in China. In this study, risk prediction models based on neural networks generally showed higher performance than logistic regression models, but they are fewer in number(*n* = 2) (26, 32), indicating that research in this area is still immature. Future research can also explore the potential contributions of other machine learning methods, such as random forests or support vector machines, to PMV prediction models. Prospective longitudinal cohort studies are ideal for the development and validation of prognostic models (16). However, the majority of studies included in our systematic review adopted a retrospective or cross-sectional cohort design, which often results in less accurate measurement of predictors and outcomes.

Although the majority of models were judged to be at high overall risk of bias according to PROBAST, they still demonstrated moderate-to-good predictive performance. Several key predictors—such as age, body mass index, prior cardiac surgery, chronic obstructive pulmonary disease, left ventricular ejection fraction, intra-aortic balloon pump use, and cardiopulmonary bypass—showed consistent associations with PMV across countries, sample sizes, and modelling approaches. This cross-study consistency, together with the presence of strong perioperative predictor–outcome relationships and relatively homogeneous single-center populations in some studies, may partly explain why moderate-to-good discrimination was observed despite methodological shortcomings. Nevertheless, high bias inevitably limits generalizability and interpretability; therefore, the findings should be applied cautiously.

To better understand the sources of this high bias, we further examined the methodological weaknesses identified by the PROBAST tool. Key analytic issues included limited sample sizes, inappropriate handling of continuous predictors and missing data, and failure to consider model overfitting. In the development of prediction models, the relationship between sample size and the frequency of outcome events (quantified as events per variable, or EPV) is fundamental. Adequate sample sizes, often determined by EPV, enhance model accuracy by reducing standard errors and narrowing confidence intervals, thereby increasing the statistical precision and robustness of prediction estimates. However, in this study, only two models meet the 20 EPV criteria (13, 27). Additionally, one model meets the minimum requirement of 10 EPV (21). Multiple studies in this review converted continuous variables—such as age, BMI, and the number of prior cardiac surgeries—into categorical forms without clearly defined cutoffs or justified rationale. While categorization may enhance usability, arbitrary classification can lead to a loss of important predictive information, thereby diminishing the model's overall accuracy (37). Additionally, two studies provided insufficient details on how missing data were managed (26, 27). Moreover, three studies excluded missing data during analysis without justification (21, 23, 33), potentially introducing selection bias. Most of the included model development studies pre-selected predictors through univariable analysis, potentially overlooking important variable interactions and increasing the risk of overfitting in multivariable models. Discrimination and calibration are crucial for evaluating prediction models. Thirteen of the included models provided discrimination metrics (AUC or C-index), indicating moderate to strong discriminatory ability However, calibration was not routinely assessed, with only eight out of fifteen studies conducting such assessments (22, 24–33). A prediction model may exhibit suboptimal performance across varying patient populations or clinical environments, so internal and external validation is essential before applying a model in clinical practice. Unfortunately, the STS risk scoring system is the only model that has been repeatedly validated by researchers in various locations and times, including validations in China, Brazil, and the USA. The meta-analysis revealed that, despite certain methodological limitations, the models demonstrated satisfactory performance. Future research should aim to expand sample sizes to meet the recommended EPV threshold, improving model precision and robustness. It is also critical to handle continuous variables appropriately, avoiding arbitrary categorization to preserve predictive power. Missing data strategies should be clearly defined to minimize selection bias.

From a broader perspective, recent years have witnessed steady progress in the prediction of PMV after CABG, with the development of diverse statistical and machine learning models, expansion of predictor domains from traditional clinical variables to novel biomarkers, and an increasing, though still limited, use of external validation. Nevertheless, several critical challenges remain: the absence of a universally accepted PMV definition hinders cross-study comparability; the majority of models are developed in single-center settings with limited geographic diversity; and translation of model outputs into actionable clinical decisions is still rare. Future research should move towards harmonizing PMV definitions, establishing large-scale, multicenter, and multinational collaborative studies, and exploring the integration of heterogeneous data sources—ranging from perioperative physiological monitoring to imaging and genomics—into predictive frameworks. Embedding validated models into electronic health records with automated risk alerts and prospectively evaluating their impact on perioperative management and patient-centered outcomes, will be key steps towards clinical adoption.

## 5 Limitations

This review has several potential limitations. Firstly, the high risk of bias observed in the majority of the included models is a matter of concern and warrants further scrutiny. This situation highlights an urgent requirement for the development of high-quality predictive models for PMV in patients undergoing CABG and emphasizes the necessity for future research to strictly adhere to methodological standards in the construction of prognostic models. Secondly, the scope of this review was constrained by language, as it included only studies published in English and Chinese. This limitation potentially excludes relevant research published in other languages or grey literature, which could introduce bias into our analysis. Thirdly, the study only assessed the statistical performance of the prediction models and did not examine their implementation in real clinical settings or their impact on decision-making. Future research should focus on prospective validation of these models in diverse clinical environments, assess their integration into routine clinical workflows, and evaluate their influence on clinical decision-making and patient outcomes. Finally, the potential for publication bias should be acknowledged. The funnel plot showed some asymmetry, although this finding should be interpreted with caution due to the small number of studies. Sensitivity analyses further demonstrated that the pooled estimates remained stable (0.6452–0.7683), indicating that the overall findings are relatively robust despite these limitations.

## 6 Conclusion

This systematic review and meta-analysis identified several factors associated with PMV for CABG patients; moreover, the performance of existing prediction models varies substantially. In clinical practice, externally validated or locally adapted models may assist early identification of high-risk patients. Future research should focus on developing robust, standardized, and widely validated models to enhance clinical applicability.

## Data Availability

The original contributions presented in the study are included in the article/Supplementary Material, further inquiries can be directed to the corresponding authors.

## References

[1] Stark B Johnson C Roth GA. Global prevalence of coronary artery disease: an update from the global burden of disease study. J Am Coll Cardiol. (2024) 83(13):2320. 10.1016/S0735-1097(24)04310-9

[2] Mensah GA Fuster V Murray CJL Roth GA Mensah GA Abate YH Global burden of cardiovascular diseases and risks, 1990–2022. J Am Coll Cardiol. (2023) 82(25):2350–473. 10.1016/j.jacc.2023.11.007 38092509 PMC7615984

[3] Vervoort D Lee G Ghandour H Guetter CR Adreak N Till BM Global cardiac surgical volume and gaps: trends, targets, and way forward. Ann Thorac Surg Short Rep. (2024) 2(2):320–4. 10.1016/j.atssr.2023.11.019 39790140 PMC11708342

[4] Rezaei Y Banar S Hadipourzadeh F Hosseini S. Mechanical ventilation during cardiopulmonary bypass improves outcomes mostly upon pleurotomy. Eur J Cardiothorac Surg. (2022) 62(3):ezac398. 10.1093/ejcts/ezac398 35900190

[5] Shahsanaei F Behrooj S Petrudi NR Khajehbahrami F. The overall prevalence and main determinants of prolonged mechanical ventilation in patients undergoing coronary artery bypass grafting: a systematic review. Heart Views. (2023) 24(4):188–93. 10.4103/heartviews.heartviews_71_23 38188710 PMC10766156

[6] Kotfis K Szylińska A Listewnik M Lechowicz K Kosiorowska M Drożdżal S Balancing intubation time with postoperative risk in cardiac surgery patients—a retrospective cohort analysis. Ther Clin Risk Manag. (2018) 14:2203–12. 10.2147/TCRM.S182333 30464493 PMC6225847

[7] Dunn H Quinn L Corbridge SJ Eldeirawi K Kapella M Collins EG. Mobilization of prolonged mechanical ventilation patients: an integrative review. Heart Lung. (2017) 46(4):221–33. 10.1016/j.hrtlng.2017.04.033 28624337 PMC6874916

[8] Ghiani A Kneidinger N Neurohr C Frank S Hinske LC Schneider C Mechanical power density predicts prolonged ventilation following double lung transplantation. Transpl Int. (2023) 36:11506. 10.3389/ti.2023.11506 37799668 PMC10548550

[9] Kumalasari RI Kosasih CE Priambodo AP. Risk factors of prolonged mechanical ventilation in post coronary artery bypass graft patients: a scoping review. J Multidiscip Healthc. (2025) 18:903–15. 10.2147/JMDH.S483973 39990641 PMC11844213

[10] Gumus F Polat A Yektas A Totoz T Bagci M Erentug V Prolonged mechanical ventilation after CABG: risk factor analysis. J Cardiothorac Vasc Anesth. (2015) 29(1):52–8. 10.1053/j.jvca.2014.09.002 25620139

[11] Yende S Wunderink R. Validity of scoring systems to predict risk of prolonged mechanical ventilation after coronary artery bypass graft surgery. Chest. (2002) 122(1):239–44. 10.1378/chest.122.1.239 12114365

[12] Liu Q Zhou Y Cao X Wang W Pan C Yichen X The impact of systemic inflammation index on prolonged mechanical ventilation after cardiac surgery: a retrospective study*.* J Cardiothorac Surg. (2025) 20(1):293. 10.1186/s13019-025-03514-7 40640849 PMC12243132

[13] Dallazen-Sartori F Albuquerque LC Guaragna JCVC da Magedanz EH Petracco JB Risk score for prolonged mechanical ventilation in coronary artery bypass grafting. Int J Cardiovasc Sci. (2021) 34:264–71.

[14] Liu Q Chen P Wang W Zhou Y Xu Y Cao X A novel scoring model for predicting prolonged mechanical ventilation in cardiac surgery patients: development and validation. Front Cardiovasc Med. (2025) 12:1573874. 10.3389/fcvm.2025.1573874 40207306 PMC11979142

[15] Damen JAA Moons KGM van Smeden M Hooft L. How to conduct a systematic review and meta-analysis of prognostic model studies. Clin Microbiol Infect. (2023) 29(4):434–40. 10.1016/j.cmi.2022.07.019 35934199 PMC9351211

[16] Li YG Huang YJ Liu G Liu ZZ Wu R. A systematic review of risk prediction models for prolonged mechanical ventilation in patients after coronary artery bypass grafting. J Nurs Sci. (2024) 39(6):58–62.

[17] Moons KGM Wolff RF Riley RD Whiting PF Westwood M Collins GS PROBAST: a tool to assess risk of bias and applicability of prediction model studies: explanation and elaboration. Ann Intern Med. (2019) 170(1):W1. 10.7326/M18-1377 30596876

[18] Debray TPA Damen JAAG Snell KIE Ensor J Hooft L Reitsma JB A guide to systematic review and meta-analysis of prediction model performance. Br Med J. (2017) 356:i6460. 10.1136/bmj.i6460 28057641

[19] Egger M Smith GD Schneider M Minder C. Bias in meta-analysis detected by a simple, graphical test. Br Med J. (1997) 315(7109):629–34. 10.1136/bmj.315.7109.629 9310563 PMC2127453

[20] Habib RH Zacharias A Engoren M. Determinants of prolonged mechanical ventilation after coronary artery bypass grafting. Ann Thorac Surg. (1996) 62(4):1164–71. 10.1016/0003-4975(96)00565-6 8823107

[21] Légaré JF Hirsch GM Buth KJ MacDougall C Sullivan JA. Preoperative prediction of prolonged mechanical ventilation following coronary artery bypass grafting. Eur J Cardio Thorac Surg. (2001) 20(5):930–6. 10.1016/S1010-7940(01)00940-X 11675177

[22] Serrano N García C Villegas J Huidobro S Henry CC Santacreu R Prolonged intubation rates after coronary artery bypass surgery and ICU risk stratification score. Chest. (2005) 128(2):595–601. 10.1378/chest.128.2.595 16100143

[23] Mu DL Wang DX. Prediction of prolonged mechanical ventilation after coronary artery bypass grafting in Chinese patients using the European system for cardiac operative risk evaluation (EuroSCORE). Med J Chin PLA. (2010) 35(12):1491–5.

[24] Ikeoka DT Fernandes VA Gebara O Garcia JCT de Barros e Silva Silva PGM Rodrigues MJ Evaluation of the society of thoracic surgeons score system for isolated coronary bypass graft surgery in a Brazilian population. Rev Bras Cir Cardiovasc: órgão of Soc Bras Cir Cardiovasc. (2014) 29(1):51–8. 10.5935/1678-9741.20140011 PMC4389475 24896163

[25] Wang TKM Li AY Ramanathan T Stewart RAH Gamble G White HD. Comparison of four risk scores for contemporary isolated coronary artery bypass grafting. Heart Lung Circ. (2014) 23(5):469–74. 10.1016/j.hlc.2013.12.001 24388496

[26] Wise ES Stonko DP Glaser ZA Garcia KL Huang JJ Kim JS Prediction of prolonged ventilation after coronary artery bypass grafting: data from an artificial neural network. Heart Surg Forum. (2017) 20(1):E7–14. 10.1532/hsf.1566 28263144

[27] O’Brien SM Feng L He X Xian Y Jacobs JP Badhwar V The society of thoracic surgeons 2018 adult cardiac surgery risk models: part 2-statistical methods and results. Ann Thorac Surg. (2018) 105(5):1419–28. 10.1016/j.athoracsur.2018.03.003 29577924

[28] Wang ZY Fu Q Wang SY Zhang B Liu JL Sun HY Predictive value of the STS risk scoring system for prolonged mechanical ventilation after off-pump coronary artery bypass grafting in a single center. Chin J Emerg Med. (2019) 39(7):642–8.

[29] Zhou SC Han HG Han JS Xu LS Wang JF Q S. Predictive value of lactate and composite models for prolonged mechanical ventilation after off-pump coronary artery bypass grafting. Med J Chin PLA. (2022) 47(5):471–8.

[30] Qi YQ Gao YK Shen XW Wu HY. Predictive performance of the Chinese coronary artery bypass graft surgery scoring system for prolonged mechanical ventilation after off-pump coronary artery bypass grafting. Prev Treat Cardio-Cerebrovasc Dis. (2022) 22(3):41–4.

[31] Liu J Zhang Y Zhang W Hu J Peng P Zhou S Prediction of vasoactive-inotropic score on prolonged mechanical ventilation in adult congenital heart disease patients after surgical treatment combined with coronary artery bypass grafting. Braz J Cardiovasc Surg. (2024) 39(3):e20230218. 10.21470/1678-9741-2023-0218 38748809 PMC11099993

[32] Mendes RG de Souza CR Machado MN Correa PR Di Thommazo-Luporini L Arena R Predicting reintubation, prolonged mechanical ventilation and death in post-coronary artery bypass graft surgery: a comparison between artificial neural networks and logistic regression models. Arch Med Sci. (2015) 11(4):756–63. 10.5114/aoms.2015.48145 26322087 PMC4548023

[33] Cislaghi F Condemi AM Corona A. Predictors of prolonged mechanical ventilation in a cohort of 5123 cardiac surgical patients. Eur J Anaesthesiol. (2009) 26(5):396–403. 10.1097/EJA.0b013e3283232c69 19276979

[34] Saleh HZ Shaw M Al-Rawi O Yates J Pullan DM Chalmers JA Outcomes and predictors of prolonged ventilation in patients undergoing elective coronary surgery. Interact Cardiovasc Thorac Surg. (2012) 15(1):51. 10.1093/icvts/ivs076 22495507 PMC3380973

[35] Zhang R Jiang H Wang H Yang Z Zhou N Gao H. Effect of advanced age on off-pump coronary artery bypass grafting. Thorac Cardiovasc Surg. (2015) 64:225–9. 10.1055/s-0035-1549273 25865780

[36] Canver CC Chanda J. Intraoperative and postoperative risk factors for respiratory failure after coronary bypass. Ann Thorac Surg. (2003) 75(3):853–7. 10.1016/S0003-4975(02)04493-4 12645706

[37] Altman DG Vergouwe Y Royston P Moons KGM. Prognosis and prognostic research: validating a prognostic model. Br Med J. (2009) 338:b605. 10.1136/bmj.b605 19477892

