# Supplementary figures and images for: Risk prediction models for prolonged mechanical ventilation following coronary artery bypass grafting surgery: a systematic review and meta-analysis
Source: Front Cardiovasc Med. 2025 Sep 12;12:1616003. doi: 10.3389/fcvm.2025.1616003 (PMC12463890; doi:10.3389/fcvm.2025.1616003)

## The Funnel Plot Results

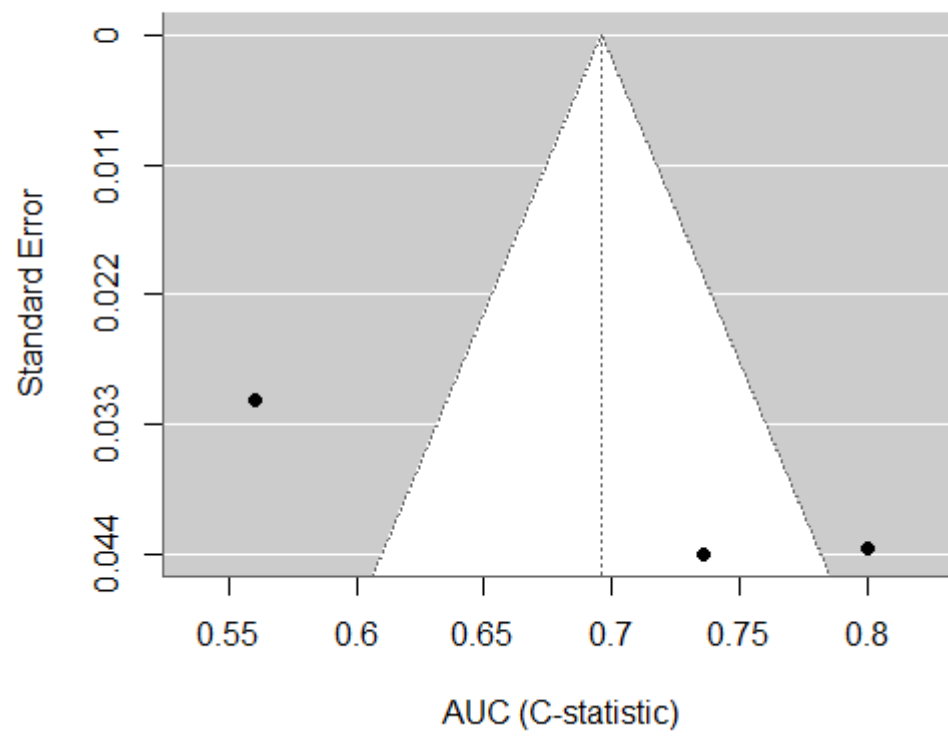

Supplement: Supplementary file 1 [file Image1.pdf]
