# Supplementary material for: Risk prediction models for prolonged mechanical ventilation following coronary artery bypass grafting surgery: a systematic review and meta-analysis
Source: Front Cardiovasc Med. 2025 Sep 12;12:1616003. doi: 10.3389/fcvm.2025.1616003 (PMC12463890; doi:10.3389/fcvm.2025.1616003)
Supplement: Supplementary file 2 [file Datasheet1.pdf]

Supplement Material 1:

Search Strategy and Search Results for each database

Literature Search Time Frame: All databases were searched from their inception to November 4, 2024.

1. PubMed

No.	Search strategy	Result
#1	"Coronary Artery Bypass"[Mesh]	57,786
#2	"Bypass Surgery, Coronary Artery"[Title/Abstract] OR "Aortocoronary Bypass*"[Title/Abstract] OR "Coronary Artery Bypass Surgery"[Title/Abstract] OR "Coronary Artery Bypass Grafting"[Title/Abstract] OR "Coronary Artery Bypasses"[Title/Abstract] OR "Bypass*, Aortocoronary"[Title/Abstract] OR "Artery Bypass*, Coronary"[Title/Abstract]	36,961
#3	#1 OR #2	69685
#4	("prolong"[Title/Abstract] OR "prolongation"[Title/Abstract] 0R "prolongations"[Title/Abstract] OR "prolonged"[Title/Abstract] OR "prolonging"[Title/Abstract] OR "prolongs"[Title/Abstract]) AND ("respiration, artificial"[MeSH Terms] OR ("respiration"[Title/Abstract] AND "artificial"[Title/Abstract]) OR "artificial respiration"[Title/Abstract] OR ("mechanical"[Title/Abstract] AND "ventilation"[Title/Abstract])OR "mechanical ventilation"[Title/Abstract]）	9138
#5	"Risk Factors"[Mesh] OR "Risk Assessment"[Mesh] OR "Early Warning Score"[Mesh] OR "Nomograms"[Mesh] OR "Logistic Models"[Mesh] OR "Regression Analysis"[Mesh] OR "Linear Models"[Mesh] OR "Models, Statistical"[Mesh] OR "Proportional Hazards Models"[Mesh] OR "Epidemiological Models"[Mesh] OR "Neural Networks, Computer"[Mesh] OR "Support Vector Machine"[Mesh] OR "Decision Trees"[Mesh] OR "Decision Support Techniques"[Mesh] OR "Machine Learning"[Mesh]	1862331

#6	Predict*[Title/Abstract] OR prognose*[Title/Abstract] OR prognostic*[Title/Abstract] OR warning*[Title/Abstract] OR model*[Title/Abstract] OR "risk instrument"[Title/Abstract] OR "risk score"[Title/Abstract] OR "Risk scoring"[Title/Abstract] OR "risk index"[Title/Abstract] OR "risk assessment instrument"[Title/Abstract] OR "risk assessment tool"[Title/Abstract] OR "risk assessment score"[Title/Abstract] OR "risk assessment measure"[Title/Abstract] OR "risk assessment measures"[Title/Abstract] OR "risk factor"[Title/Abstract] OR "risk factors"[Title/Abstract] OR "risk evaluation"[Title/Abstract] OR "Risk Appraisal"[Title/Abstract] OR "risk stratification"[Title/Abstract] OR "risk stratifications"[Title/Abstract] OR "risk-stratified"[Title/Abstract] OR "Hierarchical risk"[Title/Abstract] OR Nomogram*[Title/Abstract] OR Regression*[Title/Abstract] OR "Neural Network"[Title/Abstract] OR "Neural Networks"[Title/Abstract] OR "Support Vector Machine"[Title/Abstract] OR "Support Vector Machines"[Title/Abstract] OR "Decision Tree"[Title/Abstract] OR "Decision Trees"[Title/Abstract] OR "Machine Learning"[Title/Abstract] OR "Machine Learnings"[Title/Abstract]	7006720
#7	#5 OR #6	7750465
#8	#3 AND #4 AND #7	179

2. EMBASE

No.	Search strategy	Result
#1	'coronary artery bypass graft'/exp	93,843
#2	'aorta coronary artery bypass':ab,ti OR 'aorta coronary bypass':ab,ti OR 'aorta coronary bypass graft':ab,ti OR 'aorta coronary vein bypass':ab,ti OR 'aorta coronary vein bypass graft':ab,ti OR 'aorta coronary vein shunt':ab,ti OR 'aortic coronary artery bypass':ab,ti OR 'aortic coronary bypass':ab,ti OR 'aorticocoronary anastomosis':ab,ti OR 'aorto coronary artery bypass':ab,ti OR 'aorto coronary bypass graft':ab,ti OR 'aorto coronary vein bypass':ab,ti OR 'aortocoronary anastomosis':ab,ti OR 'aortocoronary artery bypass':ab,ti OR 'aortocoronary artery bypass graft':ab,ti OR 'aortocoronary bypass':ab,ti OR 'aortocoronary bypass graft':ab,ti OR 'aortocoronary shunt':ab,ti OR 'aortocoronary vein bypass':ab,ti OR 'aortocoronary vein bypass graft':ab,ti OR 'aortocoronary venous bypass':ab,ti OR 'aortocoronary venous bypass graft':ab,ti OR 'coronary artery bypass':ab,ti OR 'coronary artery bypass grafting':ab,ti OR 'coronary artery graft':ab,ti OR 'coronary bypass':ab,ti OR 'coronary bypass graft':ab,ti OR 'coronary bypass grafting':ab,ti OR 'coronary vein bypass graft':ab,ti OR 'coronary venous bypass graft':ab,ti OR 'coronary artery bypass graft':ab,ti	78,189
#3	#1 OR #2	118,984
#4	'artificial ventilation'/exp	314,154
#5	'artificial respiration':ab,ti OR 'artificial respiratory support':ab,ti OR 'artificial ventilatory support':ab,ti OR 'controlled respiration':ab,ti OR 'controlled ventilation':ab,ti OR 'mechanical respiration':ab,ti OR 'mechanical ventilation':ab,ti OR 'respiration, artificial':ab,ti OR 'ventilation, artificial':ab,ti OR 'artificial ventilation':ab,ti	109,743
#6	#4 OR #5	329,280
#7	prolong*:ab,ti	651,245
#8	#6 AND #7	21,535
#9	predict*:ab,ti OR prognose*:ab,ti OR prognostic*:ab,ti OR warning*:ab,ti OR model*:ab,ti OR 'risk instrument':ab,ti OR 'risk	9,071,841

	score':ab,ti OR 'risk scoring':ab,ti OR 'risk index':ab,ti OR 'risk assessment instrument':ab,ti OR 'risk assessment tool':ab,ti OR 'risk assessment score':ab,ti OR 'risk assessment measure':ab,ti OR 'risk assessment measures':ab,ti OR 'risk factor':ab,ti OR 'risk factors':ab,ti OR 'risk evaluation':ab,ti OR 'risk appraisal':ab,ti OR 'risk stratification':ab,ti OR 'risk stratifications':ab,ti OR 'risk-stratified':ab,ti OR 'hierarchical risk':ab,ti OR nomogram*:ab,ti OR regression*:ab,ti OR 'neural network':ab,ti OR 'neural networks':ab,ti OR 'support vector machine':ab,ti OR 'support vector machines':ab,ti OR 'decision tree':ab,ti OR 'decision trees':ab,ti OR 'machine learning':ab,ti OR 'machine learnings':ab,ti	
#10	'risk factor'/exp OR 'risk assessment'/exp OR 'early warning score'/exp OR 'nomogram'/exp OR 'statistical model'/exp OR 'proportional hazards model'/exp OR 'epidemiological model'/exp OR 'artificial neural network'/exp OR 'support vector machine'/exp OR 'decision tree'/exp OR 'decision support system'/exp OR 'machine learning'/exp	3,171,603
#11	#9 OR #10	10,132,735
#12	#3 AND #8 AND #11	423

3. Web of science

No.	Search strategy	Result
#1	TS=("Coronary Artery" OR "Bypass, Coronary Artery" OR "Bypass Surgery, Coronary Artery" OR "Aortocoronary Bypass*" OR "Coronary Artery Bypass Surgery" OR "Coronary Artery Bypass Grafting" OR "Coronary Artery Bypasses" OR "Bypass*, Aortocoronary" OR "Artery Bypass*, Coronary") and Preprint Citation Index (Exclude – Database)	401,982
#2	TS=(("prolong" OR "prolongation" 0R "prolongations" OR "prolonged" OR "prolonging" OR "prolongs") AND ("respiration, artificial" OR ("respiration" AND "artificial") OR "artificial respiration" OR ("mechanical" AND "ventilation")OR "mechanical ventilation"）) and Preprint Citation Index (Exclude – Database)	9,986
#3	TS=(Predict* OR prognose* OR prognostic* OR warning* OR model* OR "risk instrument" OR "risk score" OR "Risk scoring" OR "risk index" OR "risk assessment instrument" OR "risk assessment tool" OR "risk assessment score" OR "risk assessment measure" OR "risk assessment measures" OR "risk factor" OR "risk factors" OR "risk evaluation" OR "Risk Appraisal" OR "risk stratification" OR "risk stratifications" OR "risk-stratified" OR "Hierarchical risk" OR Nomogram* OR Regression* OR "Neural Network" OR "Neural Networks" OR "Support Vector Machine" OR "Support Vector Machines" OR "Decision Tree" OR "Decision Trees" OR "Machine Learning" OR "Machine Learnings") and Preprint Citation Index (Exclude – Database)	39,141,805
#4	TS=("Risk Factors" OR "Risk Assessment" OR "Early Warning Score" OR "Nomograms" OR "Logistic Models" OR "Regression Analysis" OR "Linear Models" OR "Models, Statistical" OR "Proportional Hazards Models" OR "Epidemiological Models" OR "Neural Networks, Computer" OR "Support Vector Machine" OR "Decision Trees" OR "Decision Support Techniques" OR "Machine Learning") and Preprint Citation Index (Exclude – Database)	3,812,908
#5	#1 OR #2 and Preprint Citation Index (Exclude – Database）	39,346,135
#6	#3 AND #4 AND #5	237

4.CINAHL

No.	Search strategy	Result
S1	(MH "Coronary Artery Bypass")	13,969
S2	TI (“Aortocoronary Bypass*” OR “Bypass, Coronary Arter*” OR “Bypasses Coronary Arter*” OR “CABG*” OR “Coronary Arteries Bypass*” OR “Coronary Artery Bypass Graft Surgery” OR “Coronary Artery Bypasses” OR “Coronary Bypass*”) OR AB (“Aortocoronary Bypass*” OR “Bypass, Coronary Arter*” OR “Bypasses Coronary Arter*” OR “CABG*” OR “Coronary Arteries Bypass*” OR “Coronary Artery Bypass Graft Surgery” OR “Coronary Artery Bypasses” OR “Coronary Bypass*”)	7,800
S3	S1 OR S2	16,321
S4	TI prolong* OR AB prolong*	67,611
S5	(MH "Respiration, Artificial+")	39,304
S6	TI (“Artificial Respiration” OR “Mechanical Ventilation” OR “Ventilation, Mechanical”) AND AB (“Artificial Respiration” OR “Mechanical Ventilation” OR “Ventilation, Mechanical”)	2,271
S7	S5 OR S6	39,534
S8	(S5 OR S6) AND (S4 AND S7)	2,117
S9	(MH "Risk Factors" OR "Risk Assessment" OR "Early Warning Score" OR "Nomograms" OR "Logistic Models" OR "Regression Analysis" OR "Linear Models" OR "Models, Statistical" OR "Proportional Hazards Models" OR "Epidemiological Models" OR "Neural Networks, Computer" OR "Support Vector Machine" OR "Decision Trees" OR "Decision Support Techniques" OR "Machine Learning"）	327,680
S10	TI (Predict* OR prognose* OR prognostic* OR warning* OR model* OR "risk instrument" OR "risk score" OR "Risk scoring" OR "risk index" OR "risk assessment instrument" OR "risk assessment tool" OR "risk assessment score" OR "risk assessment measure" OR "risk assessment measures" OR "risk factor" OR "risk factors" OR "risk evaluation" OR "Risk Appraisal" OR "risk stratification" OR "risk stratifications" OR	222,473

	"risk-stratified" OR "Hierarchical risk" OR Nomogram* OR Regression* OR "Neural Network" OR "Neural Networks" OR "Support Vector Machine" OR "Support Vector Machines" OR "Decision Tree" OR "Decision Trees" OR "Machine Learning" OR "Machine Learnings") AND AB (Predict* OR prognose* OR prognostic* OR warning* OR model* OR "risk instrument" OR "risk score" OR "Risk scoring" OR "risk index" OR "risk assessment instrument" OR "risk assessment tool" OR "risk assessment score" OR "risk assessment measure" OR "risk assessment measures" OR "risk factor" OR "risk factors" OR "risk evaluation" OR "Risk Appraisal" OR "risk stratification" OR "risk stratifications" OR "risk-stratified" OR "Hierarchical risk" OR Nomogram* OR Regression* OR "Neural Network" OR "Neural Networks" OR "Support Vector Machine" OR "Support Vector Machines" OR "Decision Tree" OR "Decision Trees" OR "Machine Learning" OR "Machine Learnings")	
S11	S9 OR S10	495,353
S12	S3 AND S8 AND S11	16

5. Cochrane Library

No.	Search strategy	Result
#1	MeSH descriptor: [Coronary Artery Bypass] explode all trees	6,841
#2	(“Aortocoronary Bypass” OR “Coronary Artery Bypass Grafting” OR “Coronary Artery Bypass Surgery” OR “Bypass Surgery, Coronary Artery” OR “Artery Bypass, Coronary” OR Bypass, Coronary Artery OR Bypasses, Coronary Artery OR Bypasses, Aortocoronary OR Bypass, Aortocoronary OR Coronary Artery Bypasses OR Aortocoronary Bypasses):ti,ab,kw	15,057
#3	#1 OR #2	15,068
#4	MeSH descriptor: [Respiration, Artificial] in all MeSH products	9568
#5	(“Artificial Respiration” OR “Artificial Respirations” OR “Respirations, Artificial” OR “Mechanical Ventilation” OR “Ventilations, Mechanical” OR “Ventilation, Mechanical” OR “Mechanical Ventilations”):ti,ab,kw	19,227
#6	#4 OR #5	22,817
#7	prolong*:ti,ab,kw	49843
#8	#6 AND #7	1539
#9	(Predict* OR prognose* OR prognostic* OR warning* OR model* OR "risk instrument" OR "risk score" OR "Risk scoring" OR "risk index" OR "risk assessment instrument" OR "risk assessment tool" OR "risk assessment score" OR "risk assessment measure" OR "risk assessment measures" OR "risk factor" OR "risk factors" OR "risk evaluation" OR "Risk Appraisal" OR "risk stratification" OR "risk stratifications" OR "risk-stratified" OR "Hierarchical risk" OR Nomogram* OR Regression* OR "Neural Network" OR "Neural Networks" OR "Support Vector Machine" OR "Support Vector Machines" OR "Decision Tree" OR "Decision Trees" OR "Machine Learning" OR "Machine Learnings"):ti,ab,kw	391972
#10	MeSH descriptor: [Risk Factors] explode all trees	38474
#11	MeSH descriptor: [Risk Assessment] explode all trees	13720
#12	MeSH descriptor: [Early Warning Score] explode all trees	12
#13	MeSH descriptor: [Nomograms] explode all trees	795
#14	MeSH descriptor: [Logistic Models] explode all trees	7335
#15	MeSH descriptor: [Regression Analysis] explode all trees	26404

#16	MeSH descriptor: [Linear Models] explode all trees	5487
#17	MeSH descriptor: [Models, Statistical] explode all trees	23181
#18	MeSH descriptor: [Proportional Hazards Models] explode all trees	7374
#19	MeSH descriptor: [Epidemiological Models] explode all trees	1
#20	MeSH descriptor: [Neural Networks, Computer] explode all trees	646
#21	MeSH descriptor: [Support Vector Machine] explode all trees	64
#22	MeSH descriptor: [Decision Trees] explode all trees	329
#23	MeSH descriptor: [Decision Support Techniques] explode all trees	4550
#24	MeSH descriptor: [Machine Learning] explode all trees	1023
#25	#10 OR #11 OR #12 OR #13 OR #14 OR #15 OR #16 OR #17 OR #18 OR #19 OR #20 OR #21 OR #22 OR #23 OR #24	75576
#26	#9 OR #25	399793
#27	#3 AND #8 AND #26	13

6. CKNI

No.	Search strategy	Result
#1	(Title/Abstract = "冠状动脉旁路移植术" (coronary artery bypass grafting) OR Title/Abstract = "冠状动脉搭桥术后" (post-CABG) OR Title/Abstract = "冠状动脉移植术" (coronary artery transplantation) OR Title/Abstract = "冠状动脉旁路手术" (coronary artery bypass surgery) OR Title/Abstract = "冠脉搭桥" (CABG)) AND (Title/Abstract = "风险预测模型" (risk prediction model) OR Title/Abstract = "预测模型" (prediction model) OR Title/Abstract = "预测价值" (predictive value) OR Title/Abstract = "预测研究" (prediction study)) AND (Title/Abstract = "机械通气时间" (mechanical ventilation time) OR Title/Abstract = "机械通气时间延长" (prolonged mechanical ventilation time) OR Title/Abstract = "延迟" (delay))	71

7. VIP

No.	Search strategy	Result
#1	((((All fields = "冠状动脉旁路移植术" (coronary artery bypass grafting) OR All fields = "冠状动脉搭桥术后" (post-CABG) OR All fields = "冠状动脉移植术" (coronary artery transplantation) OR All fields = "冠状动脉旁路手术" (coronary artery bypass surgery) OR All fields = "冠脉搭桥" (CABG)) AND (All fields = "风险预测模型" (risk prediction model) OR All fields = "预测模型" (prediction model) OR All fields = "预测价值" (predictive value) OR All fields = "预测研究" (prediction study))) AND (All fields = "机械通气时间" (mechanical ventilation time) OR All fields = "机械通气时间延长" (prolonged mechanical ventilation time) OR All fields = "延迟" (delay))))	29

8. Sinomed

No.	Search strategy	Result
#1	(("冠状动脉分流术"[General fields] (coronary artery shunting) OR "Coronary Artery Bypass"[General fields] OR "冠状动脉旁路"[General fields] (coronary artery bypass) OR "冠状动脉旁路手术"[General fields] (coronary artery bypass surgery) OR "主动脉冠状动脉分流术"[General fields] (aortocoronary shunting) OR "冠状动脉旁路移植术"[General fields] (coronary artery bypass grafting) OR "冠状动脉分流术"[Subject heading]) OR "冠状动脉旁路移植术 OR 冠状动脉旁路手术 OR 主动脉冠状动脉分流术"[General fields]) AND ("机械通气延长"[General fields] (prolonged mechanical ventilation) OR ("机械通气"[General fields] (mechanical ventilation) OR "人工呼吸"[General fields] (artificial respiration) OR "Artificial Respiration"[General fields] OR "呼吸，人工"[Subject heading]) OR "延迟"[General fields] (delay)) AND (("预测"[General fields] (prediction) OR "Forecasting"[General fields] OR "预测和判断"[General fields] (prediction and judgment) OR "未来"[General fields] (future) OR "未来学"[General fields] (futurology) OR "预测"[Subject heading] OR "预测模型"[General fields] (prediction model) OR "模型预测"[General fields] (model prediction) OR "预测价值"[General fields] (predictive value) OR "预测效果"[General fields] (predictive effect) OR "预测研究"[General fields] (prediction study)))	48

9. 万方

No.	Search strategy	Result
#1	All fields: ("冠状动脉旁路移植术" (coronary artery bypass grafting) OR "冠状动脉搭桥术后" (post-CABG) OR "冠状动脉移植术" (coronary artery transplantation) OR "冠状动脉旁路手术" (coronary artery bypass surgery) OR "冠脉搭桥" (CABG)) AND All fields: ("风险预测模型" (risk prediction model) OR "预测模型" (prediction model) OR "预测价值" (predictive value) OR "预测研究" (prediction study)) AND All fields: ("机械通气时间" (mechanical ventilation time) OR "机械通气时间延长" (prolonged mechanical ventilation time) OR "延迟" (delay))	79
