# Supplementary material for: Risk prediction models for prolonged mechanical ventilation following coronary artery bypass grafting surgery: a systematic review and meta-analysis
Source: Front Cardiovasc Med. 2025 Sep 12;12:1616003. doi: 10.3389/fcvm.2025.1616003 (PMC12463890; doi:10.3389/fcvm.2025.1616003)
Supplement: Supplementary file 3 [file Datasheet2.pdf]

Supplementary Material 2:

Below is the detailed PICOST.

P (Population): The population of interest includes patients aged 18 years and older who have undergone CABG.

I (Intervention): The intervention consists of published, developed, or updated risk prediction models for PMV in CABG patients.

C (Comparison): Not applicable.

O (Outcome): The outcome is defined as the duration of extended mechanical ventilation, based on the criteria employed in the included studies.

T (Time): The timing for applying the predictions is during the postoperative period following coronary artery bypass grafting.

S (Setting): The model is designed to support personalized prediction of PMV risk in the context of CABG surgery.
