# Supplementary material for: Risk prediction models for prolonged mechanical ventilation following coronary artery bypass grafting surgery: a systematic review and meta-analysis
Source: Front Cardiovasc Med. 2025 Sep 12;12:1616003. doi: 10.3389/fcvm.2025.1616003 (PMC12463890; doi:10.3389/fcvm.2025.1616003)
Supplement: Supplementary file 4 [file Datasheet3.pdf]

Supplementary Material 3

Author (year)	Final Predictors
Dallazen-Sartori et al. (2021)	6:Age ≥ 65 years, urgent/emergency surgery, BMI ≥ 30 kg/m², chronic kidney disease, COPD, cardiopulmonary bypass time ≥ 120 minutes
Légaré et al. (2001)	6:unstable angina, low ejection fraction, COPD, pre-op renal failure, female gender,advanced age
Liu et al. (2024)	3:vasoactive-inotropic score(VIS) at the end of surgery,uric acid,cardiopulmonary bypass time
Mendes et al. (2015)	10:Age,gender,weight,height, BMI, diabetes, creatinine level, cardiopulmonary bypass use, ventricular function, total number of grafts
O'Brien et al. (2018)	51:ADP usage / timing of discontinuation,Age,Alcohol consumption (drinks per week),Aortic insufficiency,Arrhythmia & type,BMI,BSA,Cardiac presentation,Chrnoc lung disease,CVD & CVA,CVD stenosis,Diabetes and control method,Ejection fraction,Family history of CAD,Glycoprotein IIb/IIIa Inhibitor w/in 24h,Heart failure class and timing,Hematocrit,Home oxygen,Hypertension,Illicit drug use,Immunosuppressive therapy,Inotrope,Left main disease,Liver disease,Mediastinal radiation,Mitral insufficiency,Myocardial infarction history/timing,Number of diseased vessels,Number of previous cardiovascular surgeries,Payor,Platelet count,Preop IABP,Previous any other cardiac intervention,Previous CAB,Previous carotid surgery,Previous ICD,PVD,Race & Ethnicity,Recent pneumonia,Recent smoker/timing,Renal function (dialysis/creatinine),Sex,Shock & ECMO & Catheter based assist device,Sleep apnea,Status,Steroid,Syncope,Time trend,Tricuspid insufficiency,Unresponsive neurologic status,White blood cell
Serrano et al. (2005)	13:Age,Body Surface Area (BSA), Number of Previous Cardiac Operations, History of Previous Operation or Angioplasty for Peripheral Vascular Disease, Preoperative Serum Creatinine Level, Preoperative Serum Albumin Level, Minutes on Cardiopulmonary Bypass, Use of an Intraaortic Balloon Pump after Cardiopulmonary Bypass, Heart Rate at ICU Admission, Cardiac Index at ICU Admission, Central Venous Pressure at ICU Admission, Arterial Bicarbonate Level at ICU Admission, Alveolar-Arterial Oxygen Pressure Gradient
Wang Ziyu et al. (2019)	4:Age ≥ 65 years，Low height, COPD, arrhythmia
Wise et al. (2017)	3:BMI, ejection fraction, cardiopulmonary bypass
Mu Dongliang et al.(2010)	4:Chronic obstructive pulmonary disease, preoperative renal dysfunction, prolonged surgery time, EuroSCORE ≥3
Zhou Shicheng et al. (2022)	5:RDW, lactate (6h post-ICU admission), pulmonary arterial pressure, NT-proBNP, IABP usage (protective factor)
Qi Yanqing et al. (2022)	6:Age, COPD, NYHA classification, LVEF < 50%, operation time, SinoSCORE
Cislaghi et al.(2007)	6:age, the duration of surgery, the presence of perioperative heart failure, the glucose level, the postoperative transfusion volume,the PaO2/FiO2 ratio
Habib et al.(1996)	7:age, smaller patient weight, New York Heart Association class IV, number of anastomoses (or grafts), fluid balance normalized to body surface area, postoperative intraaortic balloon pump use, bank blood transfusions
