# Supplementary material for: Risk prediction models for prolonged mechanical ventilation following coronary artery bypass grafting surgery: a systematic review and meta-analysis
Source: Front Cardiovasc Med. 2025 Sep 12;12:1616003. doi: 10.3389/fcvm.2025.1616003 (PMC12463890; doi:10.3389/fcvm.2025.1616003)
Supplement: Supplementary file 5 [file Datasheet4.pdf]

Supplementary Material 4

Sensitivity Analysis Results

	Study	EffectSize	CI_Lower	CI_Upper
intrcpt	Ikeoka et al.(2014)	0.6452374	0.4728456	0.8176292
intrcpt1	Wang et al.(2014)	0.7683478	0.7056326	0.8310629
intrcpt2	Wang et al.(2019)	0.6780668	0.4429016	0.9132320

Ikeoka et al. (2014): Excluding this study results in an effect size of 0.6452 with a wider confidence interval, indicating that this study has a notable impact on the overall effect size.

Wang et al. (2014): Excluding this study results in an effect size of 0.7683 with a narrower confidence interval and a higher effect size, suggesting that this study has a significant influence on the overall effect size.

Wang et al. (2019): Excluding this study results in an effect size of 0.6781 with a wider confidence interval, indicating that this study also has a notable impact on the overall effect size.
